# Proteasome targeting of proteins in Arabidopsis leaf mesophyll, epidermal and vascular tissues

**DOI:** 10.3389/fpls.2015.00376

**Published:** 2015-05-28

**Authors:** Julia Svozil, Wilhelm Gruissem, Katja Baerenfaller

**Affiliations:** Plant Biotechnology, Department of Biology, Swiss Federal Institute of Technology ZurichZurich, Switzerland

**Keywords:** protein level regulation, ubiquitylation, proteasome inhibition, leaf tissues, epidermis, mesophyll, vasculature, *Arabidopsis thaliana*

## Abstract

Protein and transcript levels are partly decoupled as a function of translation efficiency and protein degradation. Selective protein degradation via the Ubiquitin-26S proteasome system (UPS) ensures protein homeostasis and facilitates adjustment of protein abundance during changing environmental conditions. Since individual leaf tissues have specialized functions, their protein composition is different and hence also protein level regulation is expected to differ. To understand UPS function in a tissue-specific context we developed a method termed Meselect to effectively and rapidly separate *Arabidopsis thaliana* leaf epidermal, vascular and mesophyll tissues. Epidermal and vascular tissue cells are separated mechanically, while mesophyll cells are obtained after rapid protoplasting. The high yield of proteins was sufficient for tissue-specific proteome analyses after inhibition of the proteasome with the specific inhibitor Syringolin A (SylA) and affinity enrichment of ubiquitylated proteins. SylA treatment of leaves resulted in the accumulation of 225 proteins and identification of 519 ubiquitylated proteins. Proteins that were exclusively identified in the three different tissue types are consistent with specific cellular functions. Mesophyll cell proteins were enriched for plastid membrane translocation complexes as targets of the UPS. Epidermis enzymes of the TCA cycle and cell wall biosynthesis specifically accumulated after proteasome inhibition, and in the vascular tissue several enzymes involved in glucosinolate biosynthesis were found to be ubiquitylated. Our results demonstrate that protein level changes and UPS protein targets are characteristic of the individual leaf tissues and that the proteasome is relevant for tissue-specific functions.

## Introduction

Plant organs are composed of different tissues that are specialized for particular biological processes and the functionality of the organ is the sum of the functions of each of its tissue types. Just as each Arabidopsis organ has its own functional proteome map (Baerenfaller et al., 2008) we also expect that individual tissues have specific protein compositions for their specific functions. In fully grown leaves, the main leaf tissue types are epidermis, mesophyll and vasculature. In Arabidopsis, the epidermis is composed of stomata and trichomes that are embedded in the single cell layer of pavement cells, which are covered with the waxy cuticle. The adaxial and abaxial epidermal tissues enclose the palisade and spongy mesophyll, which represent the main photosynthetic capacity of the leaf. Embedded in the mesophyll tissue is the vascular tissue, which consists of phloem, xylem and cambial cells (Tsukaya, 2002).

Considering the specific functions of the different leaf tissues their protein composition would be expected to vary, however, leaf tissue-specific proteome information is currently not available. Since the correlation of absolute protein and transcript levels and their dynamic changes over time is limited (Baerenfaller et al., 2008, 2012; Walley et al., 2013), regulatory processes such as protein degradation likely influence the composition of tissue-specific proteomes. The activity of the ubiquitin-26S proteasome system (UPS) should therefore have specific signatures for individual specialized tissues. We previously identified changes in leaf protein composition after proteasome inhibition and a large number of direct UPS targets in leaves and roots (Svozil et al., 2014). However, the whole organ information does not have the necessary resolution to understand UPS function in a tissue-specific context because by homogenizing organs functional protein signatures cannot longer be attributed to individual tissues or cell types (Brandt, 2005).

To avoid this limitation and conserve specific information, different techniques for the separation of tissues or cell types have been developed. In plants, pollen and tissue culture cells can be collected effectively because they are not attached to other tissues. For example, it has been possible to obtain enough Arabidopsis pollen for a large-scale proteomics study (Grobei et al., 2009). Trichomes and root hairs are other specialized plant cells that can be easily collected for small-scale proteomics experiments (Wienkoop et al., 2004; Marks et al., 2008; Brechenmacher et al., 2009; Nestler et al., 2011; Van Cutsem et al., 2011). Microcapillaries have also been used to collect cell sap from epidermal cells (Wienkoop et al., 2004) and vascular S-cells (Koroleva and Cramer, 2011) for proteomic analyses. However, this method is restricted to accessible cell types and soluble proteins only. Alternatively, single cell or tissue types can be precisely excised from tissue sections using laser capture microdissection, for example epidermal and vascular cells of maize coleoptiles (Nakazono et al., 2003) and Arabidopsis vascular bundle cells (Schad et al., 2005). For any other approach of selecting specific cell types, the cells first need to be released from their tissue context, which in plants requires the degradation of cell walls to obtain protoplasts. Leaf mesophyll and guard cell protoplasts can be easily collected and analyzed (Zhao et al., 2008; Zhu et al., 2009). Enrichment of guard cell protoplasts expressing green fluorescent protein (GFP) has been achieved using fluorescent activating cell sorting (FACS) (Gardner et al., 2009). However, FACS of leaf protoplasts is challenging because of the chlorophyll autofluorescence that can interfere with the sorting process (Galbraith, 2007). Nevertheless, FACS in combination with enhancer trap lines expressing GFP specifically in the leaf epidermal, vasculature or guard cells has allowed enrichment of the respective cell type (Grønlund et al., 2012). FACS has also been employed for transcriptome and proteome analysis of specific cell types in Arabidopsis roots both under standard and stress conditions (Birnbaum et al., 2003, 2005; Brady et al., 2007; Dinneny et al., 2008; Petricka et al., 2012). Recently, a method of protoplasting followed by sonication and manual separation of cotyledon epidermal and vascular cells was reported for tissue-specific transcriptome analyses (Endo et al., 2014). In general, however, single cell and tissue type-specific high-throughput proteomics experiments are challenging because of the availability of material and low protein content of plants cells (Wienkoop et al., 2004; Koroleva and Cramer, 2011).

To enable tissue-specific proteome analyses we developed a rapid and effective method for the separation of the different leaf tissue types (Meselect, mechanical separation of leaf compound tissues). Here we report the specificity of the method in separating leaf mesophyll, vasculature and epidermis tissues. Using Meselect we generated tissue type-specific functional protein maps and identified responses to inhibition of the proteasome that account for the diverse composition of the leaf tissue types. Because Meselect produces a high enough yield of tissue-specific protein extracts for affinity enrichment experiments, we found several tissue-specific UPS target proteins that provide new insights into the role of protein degradation in tissue functions.

## Materials and methods

### Plant growth and inhibition of the proteasome

Seeds of *Arabidopsis thaliana* ecotype Col-0 were stratified for 2 days in darkness at 4°C and afterwards grown in short day conditions with 8 h light and 16 h darkness for 55 days at 22°C, 70% humidity The proteasome was inhibited by spraying the adaxial epidermis of leaves with 10 μM Syringolin A in 0.02% (v/v) Tween 20. As a mock control leaves were sprayed with 0.02% (v/v) Tween 20 only. The whole rosette was treated 30–60 min before the end of the night period and leaves were harvested 8 h after treatment. SylA was produced as described in Svozil et al. (2014) and was kindly provided by R. Dudler (University of Zurich, Switzerland). The experiment was performed in three biological replicates.

### Separation of the different leaf tissues with the Meselect method

After harvesting of the leaf, the TAPE sandwich method (Wu et al., 2009) was applied to remove the abaxial epidermis from the leaf. For this, the leaf was positioned between two tape stripes and the tape facing the abaxial epidermis was removed. The tape containing the abaxial epidermis was incubated for 15 min in protoplasting solution (1% cellulase Onozuka RS (Yakult, Japan), 0.25% macerozyme Onozuka R10 (Yakult, Japan), 0.4 M mannitol, 10 mM CaCl_2_, 20 mM KCl, 0.1% BSA and 20 mM MES, pH 5.7) under constant agitation at 50 rpm. During this process spongy mesophyll cells adhering to the epidermis are released into the solution, but the epidermis remains attached to the tape. The tape including epidermis was washed twice in washing buffer (154 mM NaCl, 125 mM CaCl_2_, 5 mM KCl, 5 mM glucose, and 2 mM MES, pH 5.7) and frozen in liquid nitrogen. After freezing, the epidermis could be easily removed from the tape with pre-cooled tweezers and scrapers. The epidermis was collected in a pre-cooled mortar, ground, and the tissue powder was stored at −80°C until further processing. The remainder of the leaf without the abaxial epidermis that was attached to the other tape was incubated for about 1 h in protoplast solution, which releases the palisade and spongy mesophyll cells into solution. The mesophyll protoplasts were collected by centrifugation at 200 × g for 2 min at 4°C and washed twice with washing buffer. After complete removal of the buffer, the protoplasts were frozen in liquid nitrogen and stored at −80°C until further processing. The tape with the remaining attached upper epidermal and vascular tissue was immersed in cold washing buffer. The vascular tissue network was removed with a tweezer, washed twice in washing buffer, frozen in liquid nitrogen, and stored at −80°C until further processing. The remainder of the tape with the attached adaxial epidermis was discarded because the specificity of the enrichment could not be confirmed for this tissue.

To verify the enrichment of different leaf tissues types the Arabidopsis GAL4-GFP enhancer trap lines with specific GFP expression in the epidermis (KC464), the vasculature (KC274) and the and mesophyll (JR11-2) were used (Gardner et al., 2009) (kindly provided by Alex Webb, UK). The leaves of the JR11-2 line in the C24 ecotype background were too fragile for the Meselect method. The line was therefore backcrossed four times in the Col-0 ecotype background to yield sufficiently robust leaves for the generation of the tissue type-specific protein preparations.

### Preparation of the tissue type-specific protein extracts and Western Blot analysis

For the preparation of the whole leaf and tissue type specific protein extracts the frozen plant material was ground with a mortar and pestle and proteins were extracted by incubation with SDS buffer [4% SDS, 40 mM Tris-base, 5 mM MgCl_2_, 2x protease inhibitor mix (Roche)] for 20 min at room temperature (RT). Non-soluble material was pelleted by centrifugation at RT for 10 min at 16,200 × g. The supernatant was subsequently cleared by ultracentrifugation at RT for 45 min at 100,000 × g. The SDS extract of epidermal cells was processed quickly and could not be stored, neither frozen nor at RT, since proteins will precipitate.

For mass spectrometry measurements, 50–150 μg of protein, depending on the tissue type, were subjected to SDS PAGE on 10% SDS gels. After electrophoretic separation of the proteins, the proteins were stained with Coomassie brilliant blue R250 and each lane was cut into 5 sections. Tryptic digest of the proteins and extraction of the peptides was carried out as described (Svozil et al., 2014).

For the Western Blots 15 μg of each extract were electrophoretically separated with SDS PAGE on 10% SDS gels. After blotting, the nitrocellulose membranes were cut horizontally in two halves. The lower parts were probed with α-GFP (1:1000 dilution, Roche) and α-mouse antibodies (1:5000, Roche), and the upper parts with α-HSP90 (1:3000, Agrisera) and α-rabbit (1:3000, Roche) antibodies. After detection of the immunofluorescence signal the membranes were stained with Coomassie R250.

### Affinity enrichment experiment

Frozen plant material was ground with a mortar and pestle. Mesophyll tissue cells were collected from 8 to 10 leaves each of three, vascular tissue of nine, and abaxial epidermal tissue of four plants. Proteins from mesophyll cells were extracted as described previously with a sequential extraction using native and urea buffer (Svozil et al., 2014). Proteins from vascular tissue or epidermal cells were extracted only with urea buffer. For the pre-clearing step 500 μg protein of mesophyll and vascular tissue in a total volume of 700–1200 μl and 400 μg protein of epidermal tissue in a total volume of 1800 μl were each washed with 100 μl sepharose CL4B (Sigma-Aldrich). The remaining affinity enrichment method was performed as described previously (Svozil et al., 2014).

### Mass spectrometry measurements

Mass spectrometry measurements were performed using a LTQ OrbiTrap XL mass spectrometer (Thermo Fisher) coupled to a NanoLC-AS1 (Eksigent) using electrospray ionization. For LC separation a capillary column packed with 8 cm C18 beads with a diameter of 3 μm and a pore size of 100 Å was used. Peptides were loaded on the column with a flow rate of 500 nl/min for 16 min and eluted by an increasing acetonitrile gradient from 3% acetonitrile to 50% acetonitrile for 60 min with a flow rate of 200 nl/min. One scan cycle was comprised of a survey full MS scan of spectra from m/z 300 to m/z 2000 acquired in the FT-Orbitrap with a resolution of *R* = 60,000 at m/z 400, followed by MS/MS scans of the five highest parent ions. CID was done with a target value of 1e4 in the linear trap. Collision energy was set to 28 V, Q value to 0.25, and activation time to 30 ms. Dynamic exclusion was enabled at a duration of 120 s.

### Interpretation of MS/MS spectra

The acquired raw spectra were transformed to mgf data format and searched against the TAIR10 database (28, download on January 17th, 2011) (Lamesch et al., 2012) with concatenated decoy database and supplemented with common contaminants (71,032 entries) using the Mascot algorithm (version 2.3.02) (Mascot Science). The search parameters used were: mass = monoisotopic, requirement for tryptic ends, 2 missed cleavages allowed, precursor ion tolerance = ±10 ppm, fragment ion tolerance = ±0.8 Da, variable modifications of methionine (M, PSI-MOD name: oxidation, mono Δ = 15.995) and static modifications of cysteine (C, PSI-MOD name: iodoacetamide derivatized residue, mono Δ = 57.0215). The diglycine tag that remains attached to ubiquitylated peptides after tryptic digest was not included as variable modification because we had previously observed that the identification of peptide ubiquitylation sites in complex peptide mixtures in which only a small portion of the peptides carry the diglycine tag will result in unreliable identifications (Svozil et al., 2014). Peptide spectrum assignments with ionscore >30 and expect value <0.015, except those of known contaminants, were filtered for ambiguity. Peptides matching to several proteins were excluded from further analyses. This does not apply to different splice variants of the same protein or to different loci sharing exactly the same amino acid sequence. All remaining spectrum assignments were inserted into the pep2pro database (Baerenfaller et al., 2011; Hirsch-Hoffmann et al., 2012). The false discovery rate (FDR) was calculated by dividing the number of reverse hits by the number of true hits times 100%. It was 0.55% for the tissue-type specific extracts and 1.6% for the affinity enrichments. The mass spectrometry proteomics data have been deposited to the ProteomeXchange Consortium (http://www.proteomexchange.org) via the PRIDE partner repository (Vizcaíno et al., 2013) with the dataset identifiers PXD000941 and PXD000942. The data are also available in the pep2pro database at www.pep2pro.ethz.ch.

### Statistical analyses and selection criteria

Proteins were quantified by normalized spectral counting according to Svozil et al. (2014) by calculating the expected contribution of each individual protein to the samples total peptide pool correcting the values using a normalization factor that balances for the theoretical number of tryptic peptides per protein and sample depth. The statistical analyses and selection criteria were applied to each tissue type-specific dataset separately. For the total protein extracts, proteins with a minimum of 5 spectra within a tissue type were included in the quantification. After filtering, the normalized spectral counts for the proteins in each tissue type were re-normalized by scaling the average to a value of 1. Proteins were considered changing in abundance between SylA and mock treatment if the fold-change of the average relative abundance over three biological replicates was more than 1.5 and the corresponding *p*-value in a paired *t*-test was smaller than 0.05, or if the fold change was an outlier. As outliers we considered fold changes that were higher than the upper adjacent values in boxplot statistics, which correspond to the values of the largest observation that is less than or equal to the upper quartile plus 1.5 times the length of the interquartile range. The upper adjacent values were: 2.94 for the mesophyll, 2.55 for the vasculature and 6.05 for the epidermis. Those proteins, which were identified in at least two of the three biological replicates in one treatment and not at all in the other treatment were also considered as changing in abundance. Proteins considered to be exclusively identified in a specific tissue type were detected with a minimum of 5 spectra in the respective tissue type, but not at all in the other two tissue types. In the affinity enrichment experiments we required a minimum of 5 different spectra to call a protein identified. With these data an index was calculated, where each protein identification in the UBA-domain affinity enrichment counts +1 and each identification in the sepharose control counts -1 toward the final index. As an example, an index of 2 either indicates that the protein was identified in two of the three UBA affinity enrichment replicates and never in the sepharose control, or that it was identified in all three UBA affinity enrichment replicates and only once in the sepharose control. For accepting a protein to be ubiquitylated we required that the protein had a minimum index of 2 and was either not detected at all in the sepharose background control or was at least 5-fold enriched. Analyses were done using the R algorithm (R Core Team, 2012) (Supplemental Data Sheet 1).

### GO categorization

GO categorization was performed as described before (Svozil et al., 2014) using the Ontologizer software (http://compbio.charite.de/ontologizer) (Bauer et al., 2008) in combination with the Arabidopsis annotation file considering aspect Biological Process. Annotations with GO evidence codes IEA (Inferred from Electronic Annotation) or RCA (Inferred from Reviewed Computational Analysis) were excluded from analyses. As background lists the organ-specific protein maps for leaves of the pep2pro TAIR10 wos dataset were used (Baerenfaller et al., 2011). Over-representation was assessed with the Topology-weighted method and the *p*-values were corrected for multiple testing using the Bonferroni method. GO categories with a *p* < 0.01 were considered significant.

## Results

### Effective separation of the leaf epidermal, vascular, and mesophyll tissues

**Figure 1:**
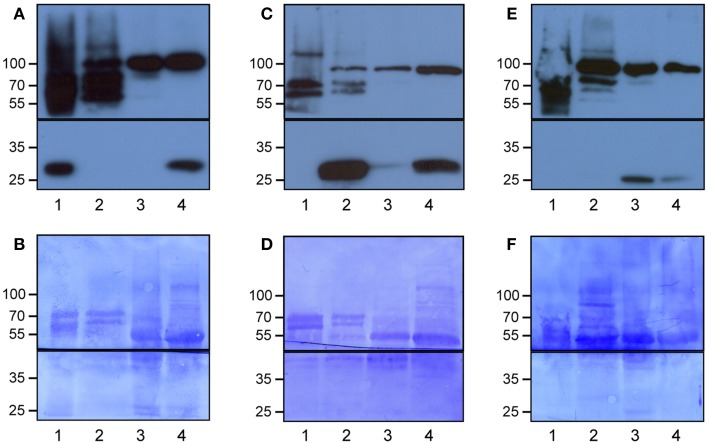
**Specific enrichment of the epidermal, vascular and mesophyll tissues using the Meselect method**. The different tissue types were separated using leaves of GAL4-GFP enhancer trap lines with GFP expression specifically in the epidermal **(A,B)**, vascular **(C,D)** or mesophyll **(E,F)** tissue. The protein blots were probed with antibodies against GFP (lower panels in **A,C,E**) and HSP90 (upper panels in **A,C,E**) as loading control, and afterwards stained with Coomassie **(B,D,F)** to show protein loading. The black lines indicate where the membranes were cut. Lane 1, epidermal tissue proteins; 2, vascular tissue proteins; 3, mesophyll tissue proteins; and 4, total leaf proteins. At the left, the molecular weight is indicated.

We developed Meselect (mechanical separation of leaf compound tissues) to specifically enrich the three main leaf tissue types. The method first utilizes the TAPE sandwich approach to separate the abaxial epidermis from the remainder of the leaf (Wu et al., 2009). Any attached mesophyll cells are released by rapid protoplasting, which leaves the epidermal cells intact. The tape is flash-frozen and the epidermal cells are collected from the tape with tweezers and scrapers. Mesophyll cells are released from the remainder of the leaf by rapid protoplasting. The vascular tissue embedded in the mesophyll tissue remains intact during protoplasting and is then isolated with a tweezer. To confirm the specificity of the enrichment we applied Meselect to leaves of Arabidopsis lines that express GFP specifically in epidermal, mesophyll and vascular tissues (Gardner et al., 2009) (Figure 1). In the tissue-type specific protein extracts of leaves from the KC464 line, which expresses GFP exclusively in the epidermis, the GFP protein was exclusively found in the epidermal protein extract but not in the protein extract of vasculature and mesophyll tissues (Figure 1A). Similarly, in the tissue type-specific protein extracts of leaves of the KC274 line, which expresses GFP only in the vasculature, GFP was specifically enriched in the vasculature protein extract (Figure 1C). We noticed small amount of GFP in the mesophyll but not the epidermal protein extract. In the JR11-2 line backcrossed with Col-0, GFP is expressed in the spongy mesophyll and GFP was detected only the mesophyll protein extract, confirming that both vasculature and epidermal protein extracts do not contain mesophyll proteins (Figure 1E). Together, the Meselect method effectively and efficiently separates the abaxial epidermal, mesophyll and vascular leaf tissues.

### Experimental design to detect tissue-specific proteins and differences in UPS targeting in different leaf tissues

**Figure 2:**
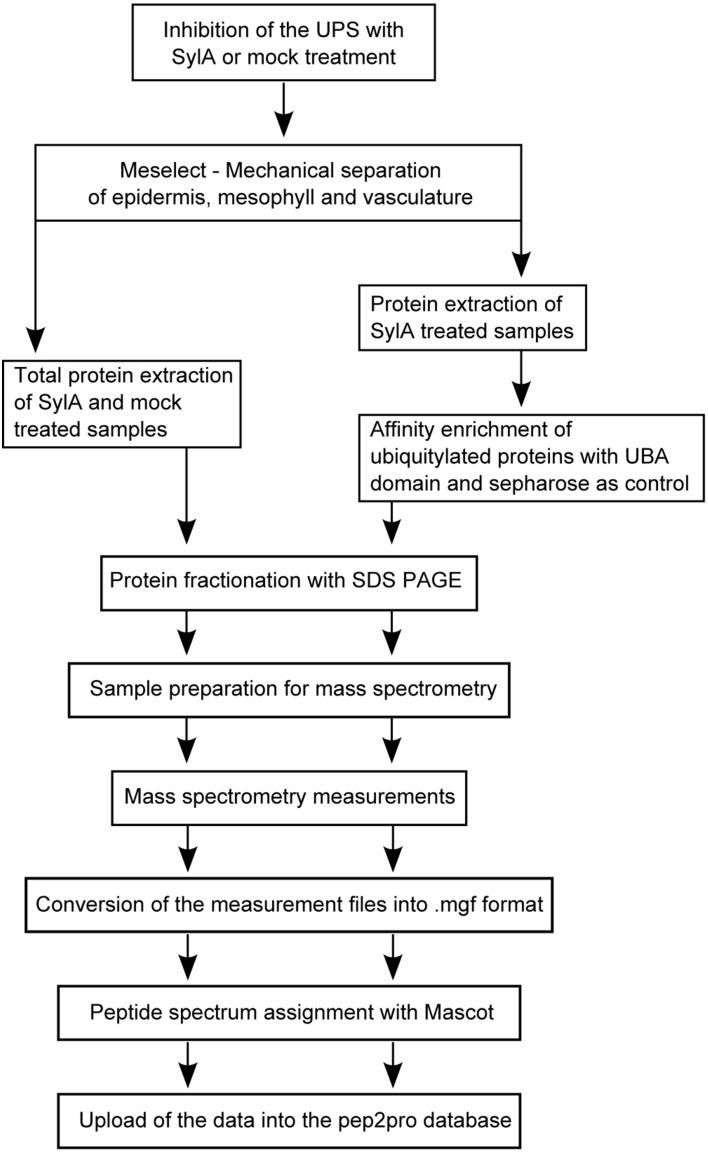
**Schematic representation of the experimental workflow**.

We adopted the workflow in Figure 2 to investigate tissue-specific differences in UPS targeting. For this we extracted total proteins from the three tissues of mock-treated leaves or leaves that were treated with Syringolin A (SylA), which specifically and effectively inhibits the UPS (Groll et al., 2008; Svozil et al., 2014). Together, we identified a total of 1799 distinct proteins and 1114 proteins that were identified with at least 5 spectra in at least one tissue (Supplemental Table 1). Of these proteins only those were considered to change in abundance if one of the following criteria was met: (i) the fold-change between SylA and mock-treated samples was >1.5 with a *p* < 0.05, (ii) the fold-change was large enough to be considered an outlier according to boxplot statistics, or (iii) the protein was identified in at least two of three biological replicates in one condition but not another condition (Table 1, Supplemental Table 2). Additionally, for tissue specificity we required that the protein was not identified in the other tissues (Table 1, Supplemental Table 3). In total, 225 distinct proteins were found that had increased and 30 that had decreased in the different leaf tissues after inhibition of the proteasome. As discussed previously, reduced protein levels could result from reduced transcription, translation or stabilization of the proteins after SylA treatment, or from proteasome-independent protein degradation (Svozil et al., 2014). In contrast, the proteins that increased after inhibition of the proteasome are likely targets of the UPS or participate in pathways that respond to SylA treatment.

**Table 1:** **Summary of the number of identified proteins in total protein extracts of epidermal, vascular and mesophyll tissues, and in ubiquitin affinity enrichments of proteins from the respective tissue types**.

**Tissue**	**Total protein extract**					**Affinity purification**	
	**Proteins identified**	**Proteins identified with ≥5 spectra**	**Exclusive for each tissue type**	**SylA**		**Ubiquitylated proteins**	**Exclusive for each tissue type**
				**Accumulation**	**decrease**		
Epidermis	1105	713	113	107	9	140	23
Vasculature	1161	730	150	61	12	353	132
Mesophyll	768	465	85	86	9	175	80
Total	1799	1114		225	30	519	

**Figure 3:**
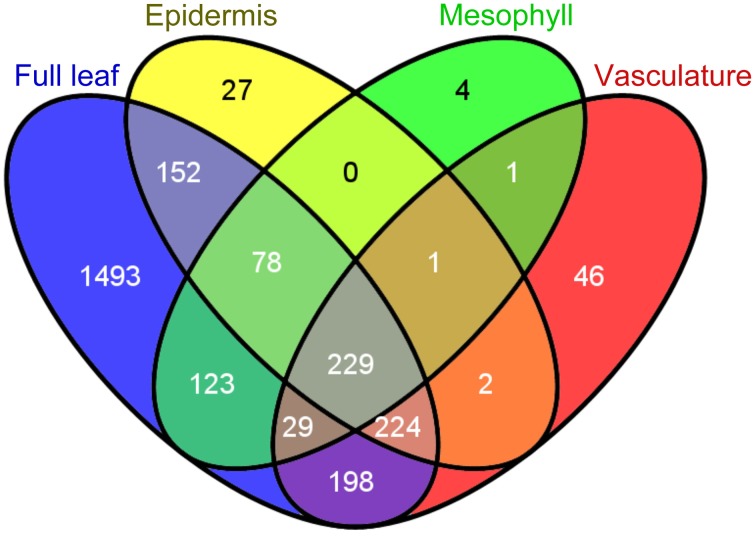
**Venn diagram displaying the number of identified proteins in a whole leaf extract (Svozil et al., 2014) and in the epidermal, mesophyll and vascular tissues**.

Using the Meselect method the protein yield in the tissue type-specific extracts was adequate for affinity enrichment of ubiquitylated proteins from SylA-treated leaves using the ubiquitin-binding UBA-domain (Sutovsky et al., 2005; Manzano et al., 2008; Svozil et al., 2014). To call a UBA affinity-enriched protein identified we required a minimum of 5 spectra for the protein in the respective tissue extract. We also calculated an index, in which each protein identification in the UBA-domain affinity enrichment counts +1 and in the sepharose control −1 for the final index. We accepted a protein to be ubiquitylated in a tissue extract if it had a minimum index of 2 and was at least 5-fold enriched over the sepharose background control. Using these criteria we identified a total of 519 ubiquitylated proteins in the three different tissues (Table 1, Supplemental Table 4). When comparing the tissue-specific ubiquitylated proteins with the reported ubiquitylated proteins identified in whole leaves (Svozil et al., 2014) we identified 161 proteins that are unique to the leaf tissue-specific dataset (Figure 3). The vascular tissue extract revealed the highest number of newly identified ubiquitylated proteins. Together, the separation of leaf tissues indeed allows the sampling of proteins that remain undetected in the whole leaf context where they are masked by proteins from abundant cell types. In the following, the datasets of proteins that are ubiquitylated and/or accumulate in the mesophyll, vasculature and epidermis after SylA treatment, as well as proteins identified in only one of the three tissue extracts, will be analyzed in detail for their tissue-type specific functions.

### Proteins identified in the tissue-specific protein extracts have specific roles in the respective tissues

#### Proteins identified exclusively in mesophyll have functions in photosynthesis

Among the 85 proteins that were only identified in the mesophyll the GO categories that are most significantly over-represented are photosynthesis, starch metabolic process and cellular polysaccharide catabolic process. The proteins in these categories comprise chloroplast proteases, components of the photosystems, as well as proteins that facilitate the incorporation of proteins into the photosystems and a kinase that phosphorylates them (Table 2). The proteins involved in starch metabolic process can be classified into two groups, starch biosynthesis and starch breakdown (Streb and Zeeman, 2012) (Table 2), and include proteins such as INOSITOL MONOPHOSPHATASE FAMILY PROTEIN (FBPase, AT1G43670) involved in cytosolic sucrose synthesis, the starch biosynthesis enzyme PHOSPHOGLUCOMUTASE (PGM, AT5G51820) and the starch breakdown enzyme PHOSPHOGLUCAN, WATER DIKINASES (GWD3, AT5G26570). This indicates that starch metabolism is mostly confined to the mesophyll tissue, although starch granules can also be observed in guard cells (Stadler et al., 2003), which are specialized cells in the epidermis.

**Table 2:** **Proteins that were exclusively identified in the mesophyll tissue and that were assigned to the significantly over-represented GO categories** ***photosynthesis*** **and** ***starch metabolic process***.

**AGI**	**Name**	**Description**	**GO category**	**Function**
AT1G50250	FtsH1	FTSH protease 1	Photosynthesis	Protease
AT5G35220	EGY1	Peptidase M50 family protein		
AT1G73060	LPA3	Low PSII accumulation 3		Incorporation of proteins in photosystems
AT4G01800	AGY1	Albino or glassy yellow 1		
AT2G45770	cpFTSY	Signal recognition particle receptor protein, chloroplast (FTSY)		
AT1G08380	PSAO	Photosystem I subunit O		Photosystem components
ATCG01010	NDHF	NADH-Ubiquinone oxidoreductase (complex I), chain 5 protein		
ATCG01110	NDHH	NAD(P)H dehydrogenase subunit H		
AT1G15980	NDH48	NDH-dependent cyclic electron flow 1		
AT5G01920	STN8	Protein kinase superfamily protein		Phosphorylation of photosystems
AT4G04020	FIB	Fibrillin		Photoinhibition
AT1G43670	FBPase	Inositol monophosphatase family protein	Photosynthesis, Starch metabolic process	Conversion triose-P to sucrose
AT5G51820	PGM	Phosphoglucomutase	Starch metabolic process	Starch biosynthesis
AT5G19220	APL1	ADP glucose pyrophosphorylase large subunit 1		
AT2G41680	NTRC	NADPH-dependent thioredoxin reductase C		
AT5G03650	BE2	Starch branching enzyme 2.2		
AT5G26570	GWD3	Catalytics;carbohydrate kinases;phosphoglucan, water dikinases		Starch breakdown
AT3G52180	SEX4	Dual specificity protein phosphatase (DsPTP1) family protein		
AT4G00490	BAM2	Beta-amylase 2		
AT4G17090	BAM3	Chloroplast beta-amylase		

#### Epidermis-specific proteins function in defense and protection

The epidermis and cuticle form the outer barrier of the leaf to the environment. Therefore it can be expected that proteins, which are involved in cuticle formation and immunoprotection are enriched in the epidermal tissue (Barel and Ginzberg, 2008; Kaspar et al., 2010; Yeats et al., 2010). Indeed, the over-represented GO categories among the 113 proteins exclusively identified in the epidermis include *innate immune response, immune response* and *immune system process*, as well as *lignin, cutin*, and *cell wall pectin biosynthetic process* (Table 3). The proteins responsible for the over-representation of immune response categories also included HSP90.1, which in addition to its chaperone function is also required for the R-gene mediated defense response (Takahashi et al., 2003). HSP90.1 was the only defense-related protein that accumulated after SylA treatment. This suggests that most of the identified defense related proteins are not targeted by the proteasome under these conditions and that the epidermal cells remain in a responsive state to counteract biotic stress. The categories *cutin biosynthetic process* and *cell wall pectin biosynthetic process* include proteins that were also identified to accumulate after inhibition of the proteasome (Table 3). These are the enzymes GLYCEROL-3-PHOSPHATE-SN-2-ACYLTRANSFERASE (GPAT4, AT1G01610) important for cutin biogenesis (Li et al., 2007; Yang et al., 2010), DEFICIENT IN CUTIN FERULATE (DCF, AT3G48720) that catalyzes the feruloylation of ω-hydroxy fatty acids, which are one type of cutin monomers (Rautengarten et al., 2012) as well as GALACTURONOSYLTRANSFERASE 1 (GAUT1, AT3G61130) and 8 (GAUT8/QUA1, AT3G25140). GAUT1 functions in an enzymatic complex that catalyzes the elongation step of homogalacturonan synthesis. GAUT8 may also function in homogalacturonan synthesis, and it was suggested to work in the hemicellulose pathway because *gaut8* mutants have a marked reduction in xylan synthase activity (Atmodjo et al., 2013). GAUT1 and GAUT7 are part of a complex that also includes KORRIGAN 1 (KOR1, AT5G49720) (Atmodjo et al., 2011) that is involved in cellulose biosynthesis and my form a link between cellulose and pectin biosynthesis and was also identified to accumulate after SylA treatment. The functions of the proteins identified exclusively in the epidermal protein extract are therefore important for establishing the special characteristics of the epidermal cell wall. Their accumulation after treatment with SylA might either point to their involvement in the general response to the treatment, or indicate that protein degradation by the UPS is one way to regulate epidermal cell wall composition.

**Table 3:** **Proteins that were exclusively identified in the epidermis and that were assigned to the significantly over-represented GO categories** ***innate immunity*** **and** ***immune response***, **as well as the categories related to cell wall biosynthesis** ***lignin biosynthetic process, cutin biosynthetic process***, **and** ***cell wall pectin biosynthetic process***.

**AGI**	**Name**	**Accumulation after SylA**	**Description**	**GO category**	**Function**
AT3G15356			Legume lectin family protein		Defense response, incompatible interaction
AT4G16260			Glycosyl hydrolase superfamily protein		
AT1G02920	GSTF7		Glutathione S-transferase 7		
AT3G04720	PR4	−	Pathogenesis-related 4		Systemic acquired resistance
AT2G44490	PEN2		Glycosyl hydrolase superfamily protein	Innate immunity, Immune response	Glucosinolate breakdown
AT5G44070	PCS1		Phytochelatin synthase 1		
AT2G26560	PLA2A		Phospholipase A 2A		Balance between cell death and defense signaling
AT5G52640	HSP90.1	+	Heat shock protein 90.1		R-gene mediated resistance
AT3G11820	SYP121		Syntaxin of plants 121		Negative regulator of defense pathways
AT4G37980	CAD7, ELI3-1, CADB1		Elicitor-activated gene 3-1	Innate immunity, Immune response, Lignin biosynthetic process	Hypersensitive response
					Lignin biosynthesis
AT4G39330	CAD9		Cinnamyl alcohol dehydrogenase 9	Lignin biosynthetic process	Lignin biosynthesis
AT1G01610	GPAT4	+	Glycerol-3-phosphate acyltransferase 4	Cutin biosynthetic process	Modification of cutin monomers
AT3G48720	DCF	+	HXXXD-type acyl-transferase family protein		
AT3G25140	QUA1, GAUT8	+	Nucleotide-diphospho-sugar transferases superfamily protein	Cell wall pectin biosynthetic process	Homogalacturonate biosynthesis
AT3G61130	GAUT1	+	Galacturonosyltransferase 1		

*In column “Accumulation after SylA” “+” indicates accumulation and “−” decreased protein amount after treatment with SylA*.

The two cinnamyl alcohol dehydrogenases CAD7 and CAD9 that were identified with 94 and 168 spectra, respectively, are involved in lignin biosynthesis (Sibout et al., 2005; Eudes et al., 2006), but their enzymatic activities were reported to be either low or non-detectable (Kim et al., 2007). GUS staining in Arabidopsis showed that the expression or localization pattern of CAD7 and CAD9 changes during development. They are exclusively expressed in the vasculature of leaves in 2 week old seedlings, but at a later developmental stage that corresponds to the age of the leaves used in our experiments, they are localized in trichomes and hydatodes (Kim et al., 2007). However, the analysis of CAD7 and CAD9 transcript accumulation over anatomy using Genevestigator (Hruz et al., 2008) revealed that their highest transcript levels were specifically detected in the translatome of the leaf epidermis (Mustroph et al., 2009). Additionally, the mRNAs of CAD7 and CAD9 and 20 additional epidermis-specific proteins were classified into the epidermis-specific translatome cluster (Mustroph et al., 2009) (Supplemental Table 3). The large overlap between exclusively identified epidermal proteins and the epidermis-specific translatome emphasizes that information on translated transcripts or proteins is important to assess tissue specific functions.

#### Comparison of leaf and root epidermal and vascular tissue proteins reveals tissue- and organ-specific processes

When comparing the identified leaf vascular tissue protein set with the root vascular tissue proteins previously reported by Petricka et al. (2012) we found an overlap of 365 proteins. In the set of 920 proteins that were identified in the root vascular tissue, but not in the leaf vascular tissue, GO biological processes such as *protein targeting to mitochondrion* and *fatty acid β-oxidation* were over-represented, corresponding well with root as a heterotrophic organ. In contrast, the 365 proteins that were identified in the leaf vascular tissue, but not in the root vascular tissue, were assigned to *glucosinolate biosynthesis*, processes related to photosynthesis, and *response to light stimulus*. The latter two were also over-represented in the 505 proteins that were identified in the leaf epidermis, but not in the root epidermis. In addition, *indole catabolic process* and *defense to fungus* were over-represented in the proteins identified in the leaf epidermis, but not in the root epidermis. In contrast, the 506 proteins that were identified in the root epidermis, but not in the leaf epidermis, were over-represented for *fatty acid β-oxidation, amino acid*, and *sterol biosynthesis*, and *thalianol metabolism*. Thalianol is a secondary metabolite that can be detected in Arabidopsis roots (Field and Osbourn, 2008). This demonstrates that the identified tissue type-specific proteins in different organs correspond to the specific functions of the respective tissue type and plant organ.

### SylA-dependent accumulating and ubiquitylated proteins in the mesophyll reveal that UPS-mediated protein degradation modulates plastid protein composition

Altogether we found that more than 50% of the ubiquitylated and more than 67% of the accumulating proteins detected in the mesophyll are predicted to be localized in the plastid according to SUBAcon in the SUBA3 database (Tanz et al., 2013). Furthermore, three of the four most significantly over-represented GO categories of the proteins that accumulated in the mesophyll after SylA treatment are *protein targeting to chloroplast, establishment of protein localization to chloroplast*, and *protein localization to chloroplast*. These categories are also over-represented in the identified ubiquitylated mesophyll proteins. Proteins of the plastid import complexes that accumulated after SylA treatment are TRANSLOCON COMPLEXES AT THE OUTER CHLOROPLAST ENVELOPE MEMBRANE 75-III (TOC75-III, AT3G46740), CHLOROPLAST SIGNAL RECOGNITION PARTICLE 54 (cpSRP54, AT5G03940), the translocase ALBINO 3 (ALB3; AT2G28800), the chloroplast chaperone CHLOROPLAST HEAT SHOCK PROTEIN 70-2 (cpHsc70-2, AT5G03940) and TRANSLOCON COMPLEXES AT THE INNER CHLOROPLAST ENVELOPE MEMBRANE 55 (TIC55, AT2G24820). TOC75-III is the channel protein in the TOC complex and TIC55 is a component of the redox regulon of the TIC complex (Kovács-Bogdán et al., 2010). Stromal Hsp70 is important for the import of precursor proteins (Su and Li, 2010), while the cpSRP consisting of cpSRP54 and cpSRP43 facilitates the passage of imported proteins through the stroma to the thylakoids (Falk and Sinning, 2010). The identified ubiquitylated mesophyll proteins included the TOC complex protein TOC159 (AT4G02510), cpSRP43 (AT2G47450) and HEAT SHOCK PROTEIN 90.5 (HSP90.5; AT2G04030). Since many of the accumulating and ubiquitylated mesophyll proteins are associated with plastid import, we compared the accumulating proteins with the set of proteins that were decreased in a TOC159-deficient mutant and are therefore putative TOC159-dependent substrates (Bischof et al., 2011). The 10 proteins in the overlap are likely proteins that are transported via the plastid import machinery and whose pre-proteins are targets of the UPS (Supplemental Table 2). Additional plastid-localized proteins that accumulated after SylA treatment are part of the plastid proteolysis system and include the proteases ORGANELLAR OLIGOPEPTIDASE (OOP; AT5G65620) and PRESEQUENCE PROTEASE 1 (PREP1; AT3G19170), as well as proteins involved in chloroplast protein degradation, namely the FtsH1 (AT1G50250), FtsH2 (AT2G30950), and FtsH8 (AT1G06430) subunits of the FtsH membrane-bound metalloprotease complex, the DEG2 (AT2G47940) subunit of the Degradation of periplasmic proteins (Deg)/high temperature requirement A (HtrA) protease, and the ClpB3 (AT5G15450) subunit of the CLP proteases. Another subunit of the CLP proteases, ClpP4 (AT5G45390) was found to be ubiquitylated. Together, the identification of the above proteins as accumulating or ubiquitylated proteins suggests that these proteins are subject to proteasome degradation and indicates that the UPS is involved in modulating the protein composition in the plastid.

### Proteins that accumulate in the epidermis after SylA treatment function in the non-cyclic flux mode of the TCA cycle

**Figure 4:**
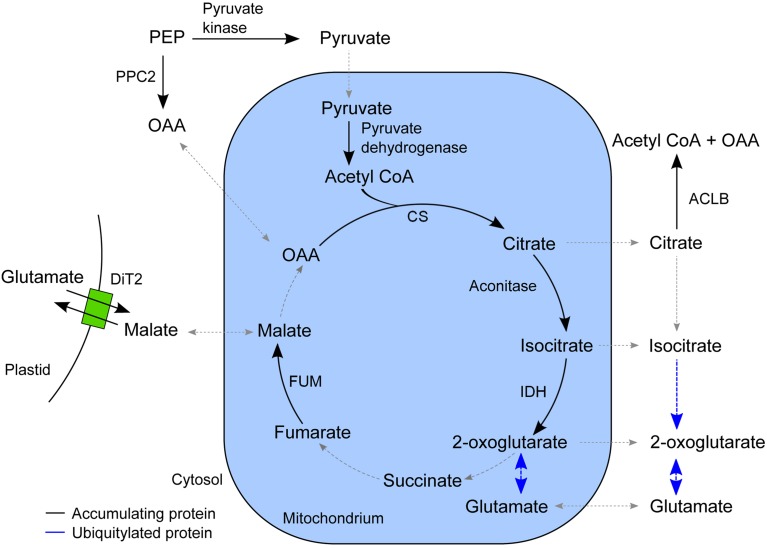
**Schematic overview of the TCA cycle and associated pathways**. The enzymes that accumulated in the epidermis after SylA treatment are indicated in their proposed subcellular compartment. Reactions for which the corresponding enzyme did not accumulate after SylA treatment are indicated by a dotted line, and those for which the corresponding enzyme was ubiquitylated by a blue line. PPC2, PEP carboxylase (AT2G42600); pyruvate kinase (AT5G56350); pyruvate dehydrogenase, E2 subunit (AT3G13930); CS, citrate synthase (AT2G44350); aconitase (AT4G35830); IDH, isocitrate dehydrogenase (AT4G35260); FUM, fumarase (AT2G47510); ACLB-2, ATP:citrate lyase, subunit B (AT5G49460) and ACLB-1 (AT3G06650); DiT2, dicarboxylate transport 2.1 (AT5G64290); OAA, oxaloacetate; PEP, phosphoenolpyruvate.

In the list of proteins that accumulated in the epidermis after SylA treatment, GO categories related to tricarboxylic acid (TCA) cycle and cell wall biosynthesis were over-represented. The plant TCA cycle produces not only reducing equivalents and ATP, but also carbon skeletons for the synthesis of amino acids (Millar et al., 2011; Nunes-Nesi et al., 2013). The non-cyclic flux mode of the TCA cycle produces 2-oxoglutarate as precursor for the amino acids glutamate and glutamine, which are the products of nitrate assimilation (Sweetlove et al., 2010). We found a remarkably high overlap in the epidermis between proteins involved in the non-cyclic flux mode of the TCA cycle and proteins that accumulated after SylA treatment (Figure 4). In addition to mitochondrial proteins that are directly involved in the TCA cycle, cytosolic proteins from connected pathways also accumulated after SylA treatment. This suggests that inhibition of the proteasome might deplete the pool of free amino acids, thus leading to increased *de novo* synthesis of glutamine and glutamate. Consistent with this hypothesis, NITRITE REDUCTASE 1 (NIR1, AT2G15620), which produces the ammonium required for the biosynthesis of glutamine and glutamate in the plastid, and DICARBOXYLATE TRANSPORT 2.1 (DiT2.1, AT5G64290), which transports glutamate from the plastid into the cytosol in exchange for malate (Renné et al., 2003), also accumulated after SylA treatment. The two mitochondrial proteins NADPH:QUINONE OXIDOREDUCTASE (AT3G27890) and a subunit of ATP synthase (AT4G29480) are involved in metabolizing NADH and FADH2 and generating ATP, respectively, and are thus part of the full TCA cycle. Their decrease after SylA treatment therefore supports the hypothesis of the TCA cycle operating in a non-flux mode in the epidermis. On the other hand, ATP:citrate lyase (ACL), which utilizes citrate to produce acetyl-CoA and thereby removes substrate from the non-cyclic flux mode TCA cycle, accumulates after SylA treatment. Cytosolic acetyl-CoA is required for the biosynthesis of a range of metabolites including elongated fatty acids (Fatland et al., 2005). Increased levels of ACL might therefore suggest an increased requirement for acetyl-CoA after SylA treatment.

None of the described enzymes of the core TCA cycle was identified to be ubiquitylated. However, CYTOSOLIC NADP+-DEPENDENT ISOCITRATE DEHYDROGENASE (CICDH, AT1G65930) and the enzymes of glutamate metabolism, GLUTAMATE DEHYDROGENASE 1 and 2 (GDH1, AT5G18170; GDH2, AT5G07440), GLUTAMATE DECARBOXYLASE 2 (AT1G65960) and GLUTAMINE SYNTHETASE 2 (AT5G35630), were ubiquitylated and are therefore putative targets of the UPS.

The expression of GDH subunits was reported to be limited to companion cells of roots and shoots for GDH1 and GDH2, or only roots for GDH3 (Fontaine et al., 2012). However, we identified GDH1 and GDH2 both in the affinity enrichments and in total extracts of epidermis and vasculature, and GDH3 was identified in the total vasculature extract. These localization data are supported by the cell-type specific leaf translatome data (Mustroph and Bailey-Serres, 2010), which indicates that the GDH subunit proteins are present in more tissues than previously reported and that their levels and the subunit composition of the GDH enzyme might be regulated by the UPS.

### Glucosinolate biosynthesis occurs primarily in the vascular tissue and is regulated by UPS targeting

**Figure 5:**
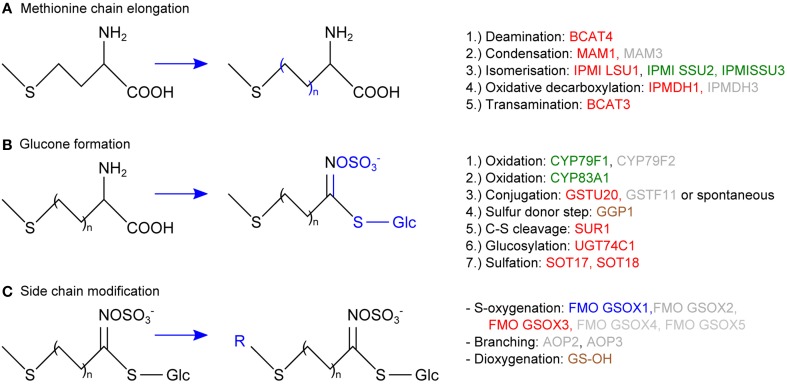
**Schematic overview of the aliphatic glucosinolate biosynthesis consisting of methionine chain elongation (A), glucone formation (B) and side chain modification (C), displaying the proteins that were exclusively identified in the vascular but not in the epidermal or mesophyll tissue extracts (green), that were exclusively identified as ubiquitylated in the vascular but not in the epidermal or mesophyll tissue extracts (blue), or both (red)**. In brown the proteins that were not exclusively identified in vasculature and in gray those that were not identified.

In vascular tissue protein extracts we identified an exceptionally high number of 353 ubiquitylated proteins (Table 1). Apart from various response pathways, the GO category *glucosinolate biosynthetic process* was over-represented in this set of proteins. This category was also over-represented in the list of proteins that were exclusively identified in the vascular but not in the epidermal or mesophyll tissue protein extracts (Table 4). Glucosinolates are derived from different amino acids, including methionine and phenylalanine. Depending on the amino acid precursor, they are assigned to different groups. Here, we focus on the biosynthesis of aliphatic glucosinolates, since the identified ubiquitylated proteins mainly catalyze reactions in this pathway. The biosynthesis of aliphatic glucosinolates involves three major steps (Grubb and Abel, 2006; Sawada et al., 2009; Sønderby et al., 2010). The first step is methionine chain elongation, which includes methionine deamination, repeated condensation, isomerization, oxidative decarboxylation and transamination. The proteins involved in this step were exclusively identified in vascular tissue protein extract and in affinity enriched ubiquitylated proteins from vascular tissue (Table 4, Figure 5). In the second step the glucone core structure is formed, which involves the incorporation of sulfur. All of the identified enzymes of the core biosynthetic process except for GGP1 were also identified exclusively in the vascular tissue protein extract or after affinity purification (Table 4, Figure 5). The third and last step in glucosinolate biosynthesis is secondary side chain modification. The identified enzymes in this process were found to be ubiquitylated exclusively in the vasculature tissue (Figure 5; Table 4). In summary, we identified most of the enzymes that catalyze the core biosynthetic steps of glucosinolate biosynthesis only in vascular tissue extracts and in affinity-enriched ubiquitylated proteins from the vascular tissue.

**Table 4:** **Proteins involved in glucosinolate metabolism identified in the tissue protein extracts and their ubiquitin affinity enrichments**.

**AGI**	**Name**	**Description**	**Process**	**Tissue protein extracts**		**Affinity enrichments**	
				**Identified**	**Only in vasculature**	**Ubiquitylated**	**Only in vasculature**
AT3G19710	BCAT4	Branched-chain aminotransferase4	Methionine chain elongation	230	x	62	x
AT5G23010	MAM1	Methylthioalkylmalate synthase 1		156	x	69	x
AT4G13430	ATLEUC1, IPMI LSU1	Isopropyl malate isomerase large subunit 1		140	x	39	x
AT2G43100	IPMI SSU2	Isopropylmalate isomerase 2		11	x		
AT3G58990	IPMI SSU3	Isopropylmalate isomerase 1		17	x		
AT5G14200	IMD1,IPMDH1	Isopropylmalate dehydrogenase 1		257	x	80	x
AT3G49680	BCAT3	Branched-chain aminotransferase3		14	x	14	x
AT1G16410	CYP79F1	Cytochrome p450 79f1	Glucone core structure formation	102	x		
AT4G13770	CYP83A1	Cytochrome P450, family 83, subfamily A, polypeptide 1		146	x		
AT1G78370	GSTU20	Glutathione S-transferase TAU 20		179	x	48	x
AT4G30530	GGP1	Gamma glutamyl peptidase 1		47		33	
AT2G20610	SUR1	Tyrosine transaminase family protein, superroot 1		182	x	60	x
AT2G31790	UGT74C1	UDP-Glycosyltransferase superfamily protein		38	x	11	x
AT1G74090	ST5b, SOT18	Desulfo-glucosinolate sulfotransferase 18		37	x	20	x
AT1G18590	ST5c, SOT17	Sulfotransferase 17		61	x	14	x
AT1G65860	FMO GSOX1	Flavin-monooxygenase glucosinolate S-oxygenase 1	Secondary side-chain modification			6	x
AT1G62560	FMO GSOX3	Flavin-monooxygenase glucosinolate S-oxygenase 3		13	x	15	x
AT2G25450	GS-OH	2-oxoglutarate (2OG) and Fe(II)-dependent oxygenase superfamily protein		57		27	x
AT2G43910	ATHOL1	Harmless to ozone layer 1	Glucosinolate degradation	16			
AT5G26000	TGG1	Thioglucoside glucohydrolase 1		1815		225	
AT5G25980	TGG2	Glucoside glucohydrolase 2		515		99	

*Proteins were assigned to the different processes in glucosinolate biosynthesis and breakdown. In columns “Identified” and “Ubiquitylated” the numbers of spectra are indicated with which the protein was identified. “x” indicates exclusive identification of the respective protein in the vascular protein extract*.

Glucosinolate breakdown is catalyzed by myrosinases and produces toxic isothiocyanates and nitriles and other reactive products that are important in plant defense. Due to the toxicity of the degradation products glucosinolates and myrosinases are located in different cell types and only brought together after tissue damage or transport of glucosinolates (Grubb and Abel, 2006; Halkier and Gershenzon, 2006; Wittstock and Burow, 2010). While we found a strong tissue-type specificity in glucosinolate biosynthesis, the localization of the two Arabidopsis myrosinases TGG1 and TGG2 is more widespread, as they were identified in all tissue-specific protein extracts with highest levels in the epidermis, and also as ubiquitylated proteins (Table 4). This broad expression pattern of the TGG1 and TGG2 proteins corresponds very well with their transcript translation data in the Arabidopsis translatome atlas (Mustroph and Bailey-Serres, 2010), although previous studies had found their expression to be limited to guard cells and phloem myrosin cell idioblasts (Koroleva et al., 2000; Andréasson et al., 2001; Husebye et al., 2002; Thangstad et al., 2004; Zhao et al., 2008).

Following myrosinase action, the S-adenosyl-l-methionine-dependent methyltransferase HARMLESS TO OZONE LAYER 1 (ATHOL1) converts thiocyanate to methylthiocyanate (Nagatoshi and Nakamura, 2009). ATHOL1 was the only protein in the glucosinolate pathway that accumulated after SylA treatment. Together, the large number of identified ubiquitylated enzymes suggests that glucosinolate synthesis and metabolism are tightly controlled by the UPS.

## Discussion

Cellular processes and responses that are analyzed in the total leaf context cannot reveal the contribution of specific tissues. We therefore developed a method to effectively separate different leaf tissues and to investigate tissue-specific processes and the response to treatment of the leaf with SylA, which inhibits the proteasome. The Meselect method described here is an effective, high-yielding method to separate mesophyll, epidermal and vascular tissues. We demonstrated that the resulting tissue type-specific protein extracts had essentially no contaminations from other tissues. Since the separation of the three tissues takes only approximately 1 h, it provides a clear advantage over other methods such as FACS. In addition, Meselect does not require the use of specific fluorescent reporter lines and is therefore compatible with experimental workflows using wild type or any transgenic plants. Meselect furthermore allows the isolation of the three different tissues from the same leaves, permitting comparisons between the tissue types and the elucidation of tissue type-specific processes. Employing the Meselect method reduced the complexity of the total leaf protein samples and therefore increased the sensitivity of the analysis. Our approach resulted in the identification of proteins that were exclusively found in the respective tissue types, of proteins that accumulated after SylA treatment and of proteins that are ubiquitylated. Based on the resulting protein lists we could assign specific processes and UPS targets to each of the three tissues.

The proteins identified in the tissue-type specific extracts are indicative of specific functions of the respective tissue. For example, proteins exclusively identified in mesophyll are related to photosynthesis as well as starch and sugar metabolic processes, and proteins in the epidermis function in defense and leaf protection. The tissue localization of other proteins that we detected in several tissue types, e.g., GDH subunits, CAD7/CAD9 and TGG1/TGG2 corresponded with the localization information in the cell-type specific leaf translatome atlas (Mustroph and Bailey-Serres, 2010). These proteins are therefore more ubiquitously expressed than previously reported. This supports our view that tissue-specific protein expression data enhance our understanding of tissue-specific processes and that tissue-type specific protein localization cannot be inferred from transcript expression data only.

Interestingly, many of the identified ubiquitylated and SylA-dependent accumulating proteins in the mesophyll are localized to the plastid. Cytosolic precursors of plastid-localized proteins are known to be degraded by the proteasome following ubiquitylation (Shen et al., 2007a,b; Lee et al., 2009, 2013). A direct interaction between the transit peptide and the 26S proteasome has been reported for certain plastid-localized proteins that may target plastid protein precursors for degradation if they are not imported (Sako et al., 2014). We therefore suggest that the ubiquitylated and SylA-dependent accumulating proteins we identified are pre-proteins that would normally be ubiquitylated and degraded in the cytosol. Furthermore, plastid import complexes themselves seem to be substrates of the UPS because E3 ligase SP1 associates with TOC complexes and mediates the ubiquitylation and degradation of TOC subunits such as TOC159 (Ling et al., 2012). We identified TOC159 and additional proteins associated with plastid transport to be ubiquitylated or accumulating after SylA treatment. UPS-mediated protein degradation might therefore modulate plastid protein composition not only by the degradation of cytosolic precursor proteins, but also by reorganization of the protein import machinery.

The UPS may also affect plastid protein homeostasis by degrading enzymes of the plastid proteolysis system involved in transit peptide cleavage and recycling, protein maturation and degradation (Adam et al., 2006; Van Wijk, 2015). After import, transit peptides of plastid-localized proteins are cleaved off in the stroma and are subsequently degraded by proteases including PREP1 and OOP (Richter and Lamppa, 1998; Kmiec and Glaser, 2012), which accumulated after inhibition of the proteasome. Protein homeostasis in the chloroplast depends on protein degradation systems of the FtsH complex, the Clp protease system, and additional proteases (Adam et al., 2006; Van Wijk, 2015). For example, the soluble protease DEG2 in cooperation with FtsH subunits is involved in repair of photosystem II by degrading the D1 reaction center protein that is damaged by light and needs to be replaced (Zaltsman et al., 2005; Kato et al., 2012). ClpB3 was shown to be essential for normal chloroplast development (Lee et al., 2007; Singh and Grover, 2010). It has also been reported that the UPS targets the precursors of the plastid proteases FtsH1 and ClpP4 for degradation (Shen et al., 2007a,b). Our finding that several proteins of the chloroplast protein degradation systems accumulated after SylA treatment suggests that the UPS is involved in plastid protein homeostasis by controlling the cytosolic accumulation of precursor proteins that themselves have proteolytic activities.

Glucosinolate biosynthesis and metabolism is an excellent example for tissue type-specific localization of proteins. Glucosinolates are sulfur-containing secondary compounds that participate in plant defense. Upon mechanical wounding of leaves, glucosinolates are hydrolysed by myrosinases that produce various toxic breakdown products, especially isothiocyanates and nitriles (Grubb and Abel, 2006; Halkier and Gershenzon, 2006). Because of the toxicity of glucosinolate breakdown products glucosinolate biosynthetic enzymes and myrosinases should not co-localize. While glucosinolates are mainly stored in vascular S-cells, myrosinases were reported to be expressed in guard cells and in idioblasts, which are located in the phloem parenchyma (Koroleva et al., 2000; Andréasson et al., 2001; Husebye et al., 2002; Thangstad et al., 2004; Grubb and Abel, 2006; Zhao et al., 2008; Wittstock and Burow, 2010). Correspondingly, we identified most of the aliphatic glucosinolate biosynthesis enzymes specifically in vascular tissue extracts. In contrast to previous reports of results from promoter:GUS expression constructs (Husebye et al., 2002; Thangstad et al., 2004), we detected the myrosinases TGG1 and TGG2 in all tissue types with highest levels in the epidermis. In Arabidopsis, vegetative rosette leaves are the major site of aliphatic, methionine-derived glucosinolate biosynthesis and storage (Andersen et al., 2013). Accordingly, we found the GO category *glucosinolate biosynthetic process* over-represented for proteins that were identified in the leaf, but not in the root vasculature (Petricka et al., 2012). As many of the glucosinolate biosynthetic enzymes were found to be ubiquitylated, the levels of these enzymes seem to be controlled by the UPS. Further experiments are required to clarify if targeted degradation regulates the tissue-type specific protein expression of the glucosinolate biosynthetic enzymes and their increased accumulation after the plant defense response has been triggered.

In the epidermis we found that many proteins of the non-cyclic flux mode of the TCA cycle accumulated after inhibition of the proteasome. In normal and carbon starvation conditions the TCA cycle drives ATP synthesis by the oxidation of respiratory substrates. Also, energy and stress signaling are thought to converge because under conditions of carbon deprivation, protein degradation is a key process for recycling cellular molecules (Baena-González and Sheen, 2008). Furthermore, UPS-mediated protein degradation has an important role in adaptation to carbon and nitrogen availability (Kang and Turano, 2003; Sato et al., 2011). However, in the non-cyclic flux mode, the TCA cycle can provide C skeletons for the biosynthesis of the primary amino acids glutamine and glutamate as well as other metabolites (Sweetlove et al., 2010). One of the functions of the proteasome system is to maintain the free amino acid pools that are needed for protein synthesis through continuous degradation of proteins (Vierstra, 1996; Zhang et al., 2014). Complete recycling of amino acids first involves the partial cleavage of proteins by the UPS followed by further degradation to free amino acids by various endo- and exopeptidases (Book et al., 2005). Inhibition of the proteasome therefore may deplete the pools of free amino acids. It was suggested that the supply of free amino acids is linked to the rate of proteolysis and that low amino acid supplies are monitored in the cell by the increasing levels of uncharged tRNAs (Vierstra, 1996). Depleted pools of free amino acids after proteasome inhibition may therefore lead to enhanced *de novo* synthesis of the primary amino acids glutamine and glutamate through activation of the non-cyclic flux mode of the TCA cycle. The mode of the TCA cycle might therefore switch between providing 2-oxoglutarate for amino acid synthesis or consuming 2-oxoglutarate for the provision of pyruvate, depending on the metabolic status of the cell and the availability of carbon, nitrogen, and amino acids. The coordination of carbon and nitrogen metabolism involves the inter-conversion of keto acids and amino acids, which is catalyzed by the enzyme glutamate dehydrogenase (GDH) (Melo-Oliveira et al., 1996; Forde and Lea, 2007; Miyashita and Good, 2008). We found that the GDH subunit proteins are present in all three leaf tissues and that they are possible targets of the UPS. In addition, CICDH that converts isocitrate to 2-oxoglutarate in the cytosol was also ubiquitylated. For darkened leaves, citrate is known to be catabolized through the TCA cycle, but in illuminated leaves, some of the produced citrate is exported from the mitochondrium to the cytosol, bypassing the mitochondrial aconitase and isocitrate dehydrogenase steps, to produce 2-oxoglutarate via cytosolic reactions (Hanning and Heldt, 1993; Lee et al., 2010). The ubiquitylation of the cytosolic enzymes for glutamate and glutamine synthesis indicates that the cytosolic part of this pathway is a direct target of the UPS. It will be interesting to determine which E3 ligases are responsible for the ubiquitylation of these proteins and what role targeted protein degradation has for regulating the mode of the TCA cycle.

In summary, the Meselect method effectively and rapidly separates epidermis, vasculature, and mesophyll tissues from the same leaf in approximately 1 h and yields high tissue amounts for proteomics. Analysis of the tissue type-specific protein localization revealed novel insights into tissue-specific processes. Moreover, the quantitative information on tissue-specific protein level changes and the types of ubiquitylated proteins that accumulated after inhibition of the proteasome by SylA has expanded our understanding of UPS-mediated control of protein accumulation in leaves. The data also support the view that protein degradation is an important mechanism for optimizing functional tissue-specific proteomes.

## Author contributions

JS and KB designed research; JS performed research; JS and KB analyzed data; JS, WG, and KB wrote the paper and approved the final version to be published.

### Conflict of interest statement

The authors declare that the research was conducted in the absence of any commercial or financial relationships that could be construed as a potential conflict of interest.

## References

[B1] Adam Z. Rudella A. van Wijk K. J. (2006). Recent advances in the study of Clp, FtsH and other proteases located in chloroplasts. Curr. Opin. Plant Biol. 9, 234–240. 10.1016/j.pbi.2006.03.010 16603408

[B2] Andersen T. G. Nour-Eldin H. H. Fuller V. L. Olsen C. E. Burow M. Halkier B. A. (2013). Integration of biosynthesis and long-distance transport establish organ-specific glucosinolate profiles in vegetative Arabidopsis. Plant Cell 25, 3133–3145. 10.1105/tpc.113.110890 23995084 PMC3784604

[B3] Andréasson E. Bolt Jørgensen L. Höglund A. S. Rask L. Meijer J. (2001). Different myrosinase and idioblast distribution in Arabidopsis and Brassica napus. Plant Physiol. 127, 1750–1763. 10.1104/pp.010334 11743118 PMC133578

[B4] Atmodjo M. A. Hao Z. Mohnen D. (2013). Evolving views of pectin biosynthesis. Annu. Rev. Plant Biol. 64, 747–779. 10.1146/annurev-arplant-042811-105534 23451775

[B5] Atmodjo M. A. Sakuragi Y. Zhu X. Burrell A. J. Mohanty S. S. Atwood J. A. III.. (2011). Galacturonosyltransferase (GAUT)1 and GAUT7 are the core of a plant cell wall pectin biosynthetic homogalacturonan:galacturonosyltransferase complex. Proc. Natl. Acad. Sci. U.S.A. 108, 20225–20230. 10.1073/pnas.1112816108 22135470 PMC3250160

[B6] Baena-González E. Sheen J. (2008). Convergent energy and stress signaling. Trends Plant Sci. 13, 474–482. 10.1016/j.tplants.2008.06.006 18701338 PMC3075853

[B7] Baerenfaller K. Grossmann J. Grobei M. A. Hull R. Hirsch-Hoffmann M. Yalovsky S.. (2008). Genome-scale proteomics reveals *Arabidopsis thaliana* gene models and proteome dynamics. Science 320, 938–941. 10.1126/science.1157956 18436743

[B8] Baerenfaller K. Hirsch-Hoffmann M. Svozil J. Hull R. Russenberger D. Bischof S.. (2011). pep2pro: a new tool for comprehensive proteome data analysis to reveal information about organ-specific proteomes in *Arabidopsis thaliana*. Integr. Biol. (Camb). 3, 225–237. 10.1039/c0ib00078g 21264403

[B9] Baerenfaller K. Massonnet C. Walsh S. Baginsky S. Bühlmann P. Hennig L.. (2012). Systems-based analysis of Arabidopsis leaf growth reveals adaptation to water deficit. Mol. Syst. Biol. 8:606. 10.1038/msb.2012.39 22929616 PMC3435506

[B10] Barel G. Ginzberg I. (2008). Potato skin proteome is enriched with plant defence components. J. Exp. Bot. 59, 3347–3357. 10.1093/jxb/ern184 18653692 PMC2529239

[B11] Bauer S. Grossmann S. Vingron M. Robinson P. N. (2008). Ontologizer 2.0–a multifunctional tool for GO term enrichment analysis and data exploration. Bioinformatics 24, 1650–1651. 10.1093/bioinformatics/btn250 18511468

[B12] Birnbaum K. Jung J. W. Wang J. Y. Lambert G. M. Hirst J. A. Galbraith D. W.. (2005). Cell type-specific expression profiling in plants via cell sorting of protoplasts from fluorescent reporter lines. Nat. Methods 2, 615–619. 10.1038/nmeth0805-615 16170893

[B13] Birnbaum K. Shasha D. E. Wang J. Y. Jung J. W. Lambert G. M. Galbraith D. W.. (2003). A gene expression map of the Arabidopsis root. Science 302, 1956–1960. 10.1126/science.1090022 14671301

[B14] Bischof S. Baerenfaller K. Wildhaber T. Troesch R. Vidi P.-A. Roschitzki B.. (2011). Plastid Proteome Assembly without Toc159: Photosynthetic Protein Import and Accumulation of N-Acetylated Plastid Precursor Proteins. Plant Cell 23, 3911–3928. 10.1105/tpc.111.092882 22128122 PMC3246318

[B15] Book A. J. Yang P. Scalf M. Smith L. M. Vierstra R. D. (2005). Tripeptidyl peptidase II. An oligomeric protease complex from Arabidopsis. Plant Physiol. 138, 1046–1057. 10.1104/pp.104.057406 15908606 PMC1150419

[B16] Brady S. M. Orlando D. A. Lee J.-Y. Wang J. Y. Koch J. Dinneny J. R.. (2007). A high-resolution root spatiotemporal map reveals dominant expression patterns. Science 318, 801–806. 10.1126/science.1146265 17975066

[B17] Brandt S. P. (2005). Microgenomics: gene expression analysis at the tissue-specific and single-cell levels. J. Exp. Bot. 56, 495–505. 10.1093/jxb/eri066 15642711

[B18] Brechenmacher L. Lee J. Sachdev S. Song Z. Nguyen T. H. N. Joshi T.. (2009). Establishment of a protein reference map for soybean root hair cells. Plant Physiol. 149, 670–682. 10.1104/pp.108.131649 19036831 PMC2633823

[B19] Dinneny J. R. Long T. A. Wang J. Y. Jung J. W. Mace D. Pointer S.. (2008). Cell identity mediates the response of Arabidopsis roots to abiotic stress. Science 320, 942–945. 10.1126/science.1153795 18436742

[B20] Endo M. Shimizu H. Nohales M. A. Araki T. Kay S. A. (2014). Tissue-specific clocks in Arabidopsis show asymmetric coupling. Nature 515, 419–422. 10.1038/nature13919 25363766 PMC4270698

[B21] Eudes A. Pollet B. Sibout R. Do C.-T. Séguin A. Lapierre C.. (2006). Evidence for a role of AtCAD 1 in lignification of elongating stems of *Arabidopsis thaliana*. Planta 225, 23–39. 10.1007/s00425-006-0326-9 16832689

[B22] Falk S. Sinning I. (2010). cpSRP43 is a novel chaperone specific for light-harvesting chlorophyll a,b-binding proteins. J. Biol. Chem. 285, 21655–21661. 10.1074/jbc.C110.132746 20498370 PMC2898393

[B23] Fatland B. L. Nikolau B. J. Wurtele E. S. (2005). Reverse genetic characterization of cytosolic acetyl-CoA generation by ATP-citrate lyase in Arabidopsis. Plant Cell 17, 182–203. 10.1105/tpc.104.026211 15608338 PMC544498

[B24] Field B. Osbourn A. E. (2008). Metabolic diversification–independent assembly of operon-like gene clusters in different plants. Science 320, 543–547. 10.1126/science.1154990 18356490

[B25] Fontaine J.-X. Terce-Laforgue T. Armengaud P. Clement G. Renou J.-P. Pelletier S.. (2012). Characterization of a NADH-dependent glutamate dehydrogenase mutant of arabidopsis demonstrates the key role of this enzyme in root carbon and nitrogen metabolism. Plant Cell 24, 4044–4065. 10.1105/tpc.112.103689 23054470 PMC3517235

[B26] Forde B. G. Lea P. J. (2007). Glutamate in plants: metabolism, regulation, and signalling. J. Exp. Bot. 58, 2339–2358. 10.1093/jxb/erm121 17578865

[B27] Galbraith D. W. (2007). Protoplast analysis using flow cytometry and sorting. Flow Cytom. Plant Cells Anal. Genes Chromosom. Genomes 231–250. 10.1002/9783527610921.ch10

[B28] Gardner M. J. Baker A. J. Assie J.-M. Poethig R. S. Haseloff J. P. Webb A. A. R. (2009). GAL4 GFP enhancer trap lines for analysis of stomatal guard cell development and gene expression. J. Exp. Bot. 60, 213–226. 10.1093/jxb/ern292 19033548 PMC3071773

[B29] Grobei M. A. Qeli E. Brunner E. Rehrauer H. Zhang R. Roschitzki B.. (2009). Deterministic protein inference for shotgun proteomics data provides new insights into Arabidopsis pollen development and function. Genome Res. 19, 1786–1800. 10.1101/gr.089060.108 19546170 PMC2765272

[B30] Groll M. Schellenberg B. Bachmann A. S. Archer C. R. Huber R. Powell T. K.. (2008). A plant pathogen virulence factor inhibits the eukaryotic proteasome by a novel mechanism. Nature 452, 755–758. 10.1038/nature06782 18401409

[B31] Grønlund J. T. Eyres A. Kumar S. Buchanan-Wollaston V. Gifford M. L. (2012). Cell specific analysis of Arabidopsis leaves using fluorescence activated cell sorting. J. Vis. Exp. 68:4214. 10.3791/4214 23070217 PMC3490320

[B32] Grubb C. D. Abel S. (2006). Glucosinolate metabolism and its control. Trends Plant Sci. 11, 89–100. 10.1016/j.tplants.2005.12.006 16406306

[B33] Halkier B. A. Gershenzon J. (2006). Biology and biochemistry of glucosinolates. Annu. Rev. Plant Biol. 57, 303–333. 10.1146/annurev.arplant.57.032905.105228 16669764

[B34] Hanning I. Heldt H. W. (1993). On the function of mitochondrial metabolism during photosynthesis in spinach (Spinacia oleracea L.) leaves (partitioning between respiration and export of redox equivalents and precursors for nitrate assimilation products). Plant Physiol. 103, 1147–1154. 10.1104/pp.103.4.1147 12232008 PMC159100

[B35] Hirsch-Hoffmann M. Gruissem W. Baerenfaller K. (2012). pep2pro: the high-throughput proteomics data processing, analysis, and visualization tool. Front. Plant Sci. 3:123. 10.3389/fpls.2012.00123 22701464 PMC3371593

[B36] Hruz T. Laule O. Szabo G. Wessendorp F. Bleuler S. Oertle L.. (2008). Genevestigator v3: a reference expression database for the meta-analysis of transcriptomes. Adv. Bioinforma. 2008:420747. 10.1155/2008/420747 19956698 PMC2777001

[B37] Husebye H. Chadchawan S. Winge P. Thangstad O. P. Bones A. M. (2002). Guard cell- and phloem idioblast-specific expression of thioglucoside glucohydrolase 1 (myrosinase) in Arabidopsis. Plant Physiol. 128, 1180–1188. 10.1104/pp.010925 11950967 PMC154246

[B38] Kang J. Turano F. J. (2003). The putative glutamate receptor 1.1 (AtGLR1.1) functions as a regulator of carbon and nitrogen metabolism in *Arabidopsis thaliana*. Proc. Natl. Acad. Sci. U.S.A. 100, 6872–6877. 10.1073/pnas.1030961100 12738881 PMC164539

[B39] Kaspar S. Matros A. Mock H.-P. (2010). Proteome and flavonoid analysis reveals distinct responses of epidermal tissue and whole leaves upon UV-B radiation of barley (Hordeum vulgare L.) seedlings. J. Proteome Res. 9, 2402–2411. 10.1021/pr901113z 20307098

[B40] Kato Y. Sun X. Zhang L. Sakamoto W. (2012). Cooperative D1 degradation in the photosystem II repair mediated by chloroplastic proteases in Arabidopsis. Plant Physiol. 159, 1428–1439. 10.1104/pp.112.199042 22698923 PMC3425188

[B41] Kim S.-J. Kim K.-W. Cho M.-H. Franceschi V. R. Davin L. B. Lewis N. G. (2007). Expression of cinnamyl alcohol dehydrogenases and their putative homologues during *Arabidopsis thaliana* growth and development: lessons for database annotations? Phytochemistry 68, 1957–1974. 10.1016/j.phytochem.2007.02.032 17467016

[B42] Kmiec B. Glaser E. (2012). A novel mitochondrial and chloroplast peptidasome, PreP. Physiol. Plant. 145, 180–186. 10.1111/j.1399-3054.2011.01531.x 21995547

[B43] Koroleva O. A. Cramer R. (2011). Single-cell proteomic analysis of glucosinolate-rich S-cells in *Arabidopsis thaliana*. Methods 54, 413–423. 10.1016/j.ymeth.2011.06.005 21708264

[B44] Koroleva O. A. Davies A. Deeken R. Thorpe M. R. Tomos A. D. Hedrich R. (2000). Identification of a new glucosinolate-rich cell type in Arabidopsis flower stalk. Plant Physiol. 124, 599–608. 10.1104/pp.124.2.599 11027710 PMC59166

[B45] Kovács-Bogdán E. Soll J. Bölter B. (2010). Protein import into chloroplasts: the Tic complex and its regulation. Biochim. Biophys. Acta 1803, 740–747. 10.1016/j.bbamcr.2010.01.015 20100520

[B46] Lamesch P. Berardini T. Z. Li D. Swarbreck D. Wilks C. Sasidharan R.. (2012). The arabidopsis information resource (TAIR): improved gene annotation and new tools. Nucleic Acids Res. 40, D1202–D1210. 10.1093/nar/gkr1090 22140109 PMC3245047

[B47] Lee C. P. Eubel H. Millar A. H. (2010). Diurnal changes in mitochondrial function reveal daily optimization of light and dark respiratory metabolism in Arabidopsis. Mol. Cell. Proteomics 9, 2125–2139. 10.1074/mcp.M110.001214 20601493 PMC2953910

[B48] Lee D. W. Jung C. Hwang I. (2013). Cytosolic events involved in chloroplast protein targeting. Biochim. Biophys. Acta 1833, 245–252. 10.1016/j.bbamcr.2012.03.006 22450030

[B49] Lee J. He K. Stolc V. Lee H. Figueroa P. Gao Y.. (2007). Analysis of transcription factor HY5 genomic binding sites revealed its hierarchical role in light regulation of development. Plant Cell 19, 731–749. 10.1105/tpc.106.047688 17337630 PMC1867377

[B50] Lee S. Lee D. W. Lee Y. Mayer U. Stierhof Y.-D. Lee S.. (2009). Heat shock protein cognate 70-4 and an E3 ubiquitin ligase, CHIP, mediate plastid-destined precursor degradation through the ubiquitin-26S proteasome system in Arabidopsis. Plant Cell 21, 3984–4001. 10.1105/tpc.109.071548 20028838 PMC2814507

[B51] Li Y. Beisson F. Koo A. J. K. Molina I. Pollard M. Ohlrogge J. (2007). Identification of acyltransferases required for cutin biosynthesis and production of cutin with suberin-like monomers. Proc. Natl. Acad. Sci. U.S.A. 104, 18339–18344. 10.1073/pnas.0706984104 17991776 PMC2084344

[B52] Ling Q. Huang W. Baldwin A. Jarvis P. (2012). Chloroplast biogenesis is regulated by direct action of the ubiquitin-proteasome system. Science 338, 655–659. 10.1126/science.1225053 23118188

[B53] Manzano C. Abraham Z. López-Torrejón G. Del Pozo J. C. (2008). Identification of ubiquitinated proteins in Arabidopsis. Plant Mol. Biol. 68, 145–158. 10.1007/s11103-008-9358-9 18535787

[B54] Marks M. D. Betancur L. Gilding E. Chen F. Bauer S. Wenger J. P.. (2008). A new method for isolating large quantities of Arabidopsis trichomes for transcriptome, cell wall and other types of analyses. Plant J. 56, 483–492. 10.1111/j.1365-313X.2008.03611.x 18643981

[B55] Melo-Oliveira R. Oliveira I. C. Coruzzi G. M. (1996). Arabidopsis mutant analysis and gene regulation define a nonredundant role for glutamate dehydrogenase in nitrogen assimilation. Proc. Natl. Acad. Sci. U.S.A. 93, 4718–4723. 10.1073/pnas.93.10.4718 8643469 PMC39345

[B56] Millar A. H. Whelan J. Soole K. L. Day D. A. (2011). Organization and regulation of mitochondrial respiration in plants. Annu. Rev. Plant Biol. 62, 79–104. 10.1146/annurev-arplant-042110-103857 21332361

[B57] Miyashita Y. Good A. G. (2008). NAD(H)-dependent glutamate dehydrogenase is essential for the survival of *Arabidopsis thaliana* during dark-induced carbon starvation. J. Exp. Bot. 59, 667–680. 10.1093/jxb/erm340 18296429

[B58] Mustroph A. Bailey-Serres J. (2010). The Arabidopsis translatome cell-specific mRNA atlas: Mining suberin and cutin lipid monomer biosynthesis genes as an example for data application. Plant Signal. Behav. 5, 320–324. 10.4161/psb.5.3.11187 20220312 PMC2881290

[B59] Mustroph A. Zanetti M. E. Jang C. J. H. Holtan H. E. Repetti P. P. Galbraith D. W.. (2009). Profiling translatomes of discrete cell populations resolves altered cellular priorities during hypoxia in Arabidopsis. Proc. Natl. Acad. Sci. U.S.A. 106, 18843–18848. 10.1073/pnas.0906131106 19843695 PMC2764735

[B60] Nagatoshi Y. Nakamura T. (2009). Arabidopsis HARMLESS TO OZONE LAYER protein methylates a glucosinolate breakdown product and functions in resistance to Pseudomonas syringae pv. maculicola. J. Biol. Chem. 284, 19301–19309. 10.1074/jbc.M109.001032 19419967 PMC2740555

[B61] Nakazono M. Qiu F. Borsuk L. A. Schnable P. S. (2003). Laser-capture microdissection, a tool for the global analysis of gene expression in specific plant cell types: identification of genes expressed differentially in epidermal cells or vascular tissues of maize. Plant Cell 15, 583–596. 10.1105/tpc.008102 12615934 PMC150015

[B62] Nestler J. Schütz W. Hochholdinger F. (2011). Conserved and unique features of the maize (Zea mays L.) root hair proteome. J. Proteome Res. 10, 2525–2537. 10.1021/pr200003k 21417484

[B63] Nunes-Nesi A. Araújo W. L. Obata T. Fernie A. R. (2013). Regulation of the mitochondrial tricarboxylic acid cycle. Curr. Opin. Plant Biol. 16, 335–343. 10.1016/j.pbi.2013.01.004 23462640

[B64] Petricka J. J. Schauer M. A. Megraw M. Breakfield N. W. Thompson J. W. Georgiev S.. (2012). The protein expression landscape of the Arabidopsis root. Proc. Natl. Acad. Sci. U.S.A. 109, 6811–6818. 10.1073/pnas,0.1202546109 22447775 PMC3344980

[B65] R Core Team (2012). R: A Language and Environment for Statistical Computing. Available online at: http://www.r-project.org

[B66] Rautengarten C. Ebert B. Ouellet M. Nafisi M. Baidoo E. E. K. Benke P.. (2012). Arabidopsis deficient in cutin ferulate encodes a transferase required for feruloylation of ω-hydroxy fatty acids in cutin polyester. Plant Physiol. 158, 654–665. 10.1104/pp.111.187187 22158675 PMC3271757

[B67] Renné P. Dressen U. Hebbeker U. Hille D. Flügge U.-I. Westhoff P.. (2003). The Arabidopsis mutant dct is deficient in the plastidic glutamate/malate translocator DiT2. Plant J. 35, 316–331. 10.1046/j.1365-313X.2003.01806.x 12887583

[B68] Richter S. Lamppa G. K. (1998). A chloroplast processing enzyme functions as the general stromal processing peptidase. Proc. Natl. Acad. Sci. U.S.A. 95, 7463–7468. 10.1073/pnas.95.13.7463 9636172 PMC22651

[B69] Sako K. Yanagawa Y. Kanai T. Sato T. Seki M. Fujiwara M.. (2014). Proteomic analysis of the 26S proteasome reveals its direct interaction with transit peptides of plastid protein precursors for their degradation. J. Proteome Res. 13, 3223–3230. 10.1021/pr401245g 24846764

[B70] Sato T. Maekawa S. Yasuda S. Yamaguchi J. (2011). Carbon and nitrogen metabolism regulated by the ubiquitin-proteasome system. Plant Signal. Behav. 6, 1465–1468. 10.4161/psb.6.10.17343 21897122 PMC3256372

[B71] Sawada Y. Kuwahara A. Nagano M. Narisawa T. Sakata A. Saito K.. (2009). Omics-based approaches to methionine side chain elongation in Arabidopsis: characterization of the genes encoding methylthioalkylmalate isomerase and methylthioalkylmalate dehydrogenase. Plant Cell Physiol. 50, 1181–1190. 10.1093/pcp/pcp079 19493961 PMC2709551

[B72] Schad M. Lipton M. S. Giavalisco P. Smith R. D. Kehr J. (2005). Evaluation of two-dimensional electrophoresis and liquid chromatography–tandem mass spectrometry for tissue-specific protein profiling of laser-microdissected plant samples. Electrophoresis 26, 2729–2738. 10.1002/elps.200410399 15971193

[B73] Shen G. Adam Z. Zhang H. (2007a). The E3 ligase AtCHIP ubiquitylates FtsH1, a component of the chloroplast FtsH protease, and affects protein degradation in chloroplasts. Plant J. 52, 309–321. 10.1111/j.1365-313X.2007.03239.x 17714429

[B74] Shen G. Yan J. Pasapula V. Luo J. He C. Clarke A. K.. (2007b). The chloroplast protease subunit ClpP4 is a substrate of the E3 ligase AtCHIP and plays an important role in chloroplast function. Plant J. 49, 228–237. 10.1111/j.1365-313X.2006.02963.x 17241447

[B75] Sibout R. Eudes A. Mouille G. Pollet B. Lapierre C. Jouanin L.. (2005). CINNAMYL ALCOHOL DEHYDROGENASE-C and -D are the primary genes involved in lignin biosynthesis in the floral stem of Arabidopsis. Plant Cell 17, 2059–2076. 10.1105/tpc.105.030767 15937231 PMC1167552

[B76] Singh A. Grover A. (2010). Plant Hsp100/ClpB-like proteins: poorly-analyzed cousins of yeast ClpB machine. Plant Mol. Biol. 74, 395–404. 10.1007/s11103-010-9682-8 20811767

[B77] Sønderby I. E. Geu-Flores F. Halkier B. A. (2010). Biosynthesis of glucosinolates–gene discovery and beyond. Trends Plant Sci. 15, 283–290. 10.1016/j.tplants.2010.02.005 20303821

[B78] Stadler R. Büttner M. Ache P. Hedrich R. Ivashikina N. Melzer M.. (2003). Diurnal and light-regulated expression of AtSTP1 in guard cells of Arabidopsis. Plant Physiol. 133, 528–537. 10.1104/pp.103.024240 12972665 PMC219029

[B78a] Streb S. Zeeman S. C. (2012). Starch metabolism in Arabidopsis. Arab. B. 10:e0160 10.1199/tab.0160 PMC3527087 23393426

[B79] Su P.-H. Li H. (2010). Stromal Hsp70 is important for protein translocation into pea and Arabidopsis chloroplasts. Plant Cell 22, 1516–1531. 10.1105/tpc.109.071415 20484004 PMC2899880

[B80] Sutovsky P. Manandhar G. Laurincik J. Letko J. Caamaño J. N. Day B. N.. (2005). Expression and proteasomal degradation of the major vault protein (MVP) in mammalian oocytes and zygotes. Reproduction 129, 269–282. 10.1530/rep.1.00291 15749954

[B81] Svozil J. Hirsch-Hoffmann M. Dudler R. Gruissem W. Baerenfaller K. (2014). Protein abundance changes and ubiquitylation targets identified after inhibition of the proteasome with Syringolin A. Mol. Cell. Proteomics 13, 1523–1536. 10.1074/mcp.M113.036269 24732913 PMC4047471

[B82] Sweetlove L. J. Beard K. F. M. Nunes-Nesi A. Fernie A. R. Ratcliffe R. G. (2010). Not just a circle: flux modes in the plant TCA cycle. Trends Plant Sci. 15, 462–470. 10.1016/j.tplants.2010.05.006 20554469

[B83] Takahashi A. Casais C. Ichimura K. Shirasu K. (2003). HSP90 interacts with RAR1 and SGT1 and is essential for RPS2-mediated disease resistance in Arabidopsis. Proc. Natl. Acad. Sci. U.S.A. 100, 11777–11782. 10.1073/pnas.2033934100 14504384 PMC208834

[B84] Tanz S. K. Castleden I. Hooper C. M. Vacher M. Small I. Millar H. A. (2013). SUBA3: a database for integrating experimentation and prediction to define the SUBcellular location of proteins in Arabidopsis. Nucleic Acids Res. 41, D1185–D1191. 10.1093/nar/gks1151 23180787 PMC3531127

[B85] Thangstad O. P. Gilde B. Chadchawan S. Seem M. Husebye H. Bradley D.. (2004). Cell specific, cross-species expression of myrosinases in Brassica napus, *Arabidopsis thaliana* and Nicotiana tabacum. Plant Mol. Biol. 54, 597–611. 10.1023/B:PLAN.0000038272.99590.10 15316292

[B86] Tsukaya H. (2002). Leaf development. Arab. B. 1:e0072 10.1199/tab.0072 PMC3243299 22303217

[B87] Van Cutsem E. Simonart G. Degand H. Faber A.-M. Morsomme P. Boutry M. (2011). Gel-based and gel-free proteomic analysis of Nicotiana tabacum trichomes identifies proteins involved in secondary metabolism and in the (a)biotic stress response. Proteomics 11, 440–454. 10.1002/pmic.201000356 21268273

[B88] Van Wijk K. J. (2015). Protein maturation and proteolysis in plant plastids, mitochondria, and peroxisomes. Annu. Rev. Plant Biol. 66, 75–111. 10.1146/annurev-arplant-043014-115547 25580835

[B89] Vierstra R. D. (1996). Proteolysis in plants: mechanisms and functions. Plant Mol. Biol. 32, 275–302. 10.1007/BF00039386 8980483

[B90] Vizcaíno J. A. Côté R. G. Csordas A. Dianes J. A. Fabregat A. Foster J. M.. (2013). The PRoteomics IDEntifications (PRIDE) database and associated tools: status in 2013. Nucleic Acids Res. 41, D1063–D1069. 10.1093/nar/gks1262 23203882 PMC3531176

[B91] Walley J. W. Shen Z. Sartor R. Wu K. J. Osborn J. Smith L. G.. (2013). Reconstruction of protein networks from an atlas of maize seed proteotypes. Proc. Natl. Acad. Sci. U.S.A. 110, E4808–E4817. 10.1073/pnas.1319113110 24248366 PMC3856832

[B92] Wienkoop S. Zoeller D. Ebert B. Simon-Rosin U. Fisahn J. Glinski M.. (2004). Cell-specific protein profiling in *Arabidopsis thaliana* trichomes: identification of trichome-located proteins involved in sulfur metabolism and detoxification. Phytochemistry 65, 1641–1649. 10.1016/j.phytochem.2004.03.026 15276459

[B93] Wittstock U. Burow M. (2010). Glucosinolate breakdown in arabidopsis: mechanism, regulation and biological significance. Arab. B. 8:e0134. 10.1199/tab.0134 22303260 PMC3244901

[B94] Wu F.-H. Shen S.-C. Lee L.-Y. Lee S.-H. Chan M.-T. Lin C.-S. (2009). Tape-arabidopsis sandwich - a simpler arabidopsis protoplast isolation method. Plant Methods 5:16. 10.1186/1746-4811-5-16 19930690 PMC2794253

[B95] Yang W. Pollard M. Li-Beisson Y. Beisson F. Feig M. Ohlrogge J. (2010). A distinct type of glycerol-3-phosphate acyltransferase with sn-2 preference and phosphatase activity producing 2-monoacylglycerol. Proc. Natl. Acad. Sci. U.S.A. 107, 12040–12045. 10.1073/pnas.0914149107 20551224 PMC2900678

[B96] Yeats T. H. Howe K. J. Matas A. J. Buda G. J. Thannhauser T. W. Rose J. K. C. (2010). Mining the surface proteome of tomato (Solanum lycopersicum) fruit for proteins associated with cuticle biogenesis. J. Exp. Bot. 61, 3759–3771. 10.1093/jxb/erq194 20571035 PMC2921210

[B97] Zaltsman A. Ori N. Adam Z. (2005). Two types of FtsH protease subunits are required for chloroplast biogenesis and Photosystem II repair in Arabidopsis. Plant Cell 17, 2782–2790. 10.1105/tpc.105.035071 16126834 PMC1242272

[B98] Zhang Y. Nicholatos J. Dreier J. R. Ricoult S. J. H. Widenmaier S. B. Hotamisligil G. S.. (2014). Coordinated regulation of protein synthesis and degradation by mTORC1. Nature 513, 440–443. 10.1038/nature13492 25043031 PMC4402229

[B99] Zhao Z. Zhang W. Stanley B. A. Assmann S. M. (2008). Functional proteomics of *Arabidopsis thaliana* guard cells uncovers new stomatal signaling pathways. Plant Cell 20, 3210–3226. 10.1105/tpc.108.063263 19114538 PMC2630442

[B100] Zhu M. Dai S. McClung S. Yan X. Chen S. (2009). Functional differentiation of Brassica napus guard cells and mesophyll cells revealed by comparative proteomics. Mol. Cell Proteomics 8, 752–766. 10.1074/mcp.M800343-MCP200 19106087 PMC2667361

